# Professionalism and Empathy Assessment in Undergraduate Medical Students: A Systematic Review

**DOI:** 10.1007/s40670-026-02752-1

**Published:** 2026-05-12

**Authors:** Noor Rejjal

**Affiliations:** Office of Educational Program, McGovern Medical School, University of Texas Health Science Center at Houston, Houston, USA

**Keywords:** Professionalism, Empathy, Undergraduate medical education, Medical students, Assessment, Systematic review, Programmatic assessment

## Abstract

**Background:**

Professionalism and empathy are core competencies in undergraduate medical education, but their assessment remains challenging because these domains are multidimensional and are measured using diverse methods. Existing reviews have often examined empathy or professionalism separately, with less attention to how different assessment modalities capture related but distinct constructs.

**Methods:**

A systematic review was conducted following PRISMA guidance to examine literature published from 2000 to 2025 on assessment of professionalism and empathy in undergraduate medical students. Included studies evaluated self-report instruments, observer-based assessments, patient- or standardized patient–perceived measures, simulation-based methods, reflective portfolios, and emerging technology-enhanced approaches. Because of substantial heterogeneity in study design, outcomes, and assessment modalities, findings were synthesized narratively by assessment type and target construct.

**Results:**

Thirty-five studies met inclusion criteria. Self-report instruments, particularly the Jefferson Scale of Empathy and related professionalism attitude scales, were the most commonly studied and showed the strongest psychometric structure for assessing empathic orientation and professional attitudes. Observer-based assessments, including faculty ratings, the Professionalism Mini-Evaluation Exercise, and multisource feedback, provided evidence of enacted behavior in authentic settings but were more vulnerable to rater effects and contextual variability. Patient- and standardized patient–based measures captured perceived empathy within specific encounters, while OSCEs and situational judgement tests offered greater standardization for assessing communication, ethical reasoning, and professional judgment. Reflective portfolios contributed insight into professional identity formation and developmental growth. Across studies, convergence between modalities was limited, suggesting that these tools assess overlapping but nonidentical constructs rather than a single unified trait. Overall, the literature supports a multimodal approach in which multiple low- and moderate-stakes assessments are interpreted together.

**Conclusions:**

Professionalism and empathy assessment in undergraduate medical education is best understood as a multi-construct, multimethod challenge. No single instrument adequately captures these competencies. A programmatic assessment approach that combines complementary methods over time is therefore more educationally and psychometrically defensible than reliance on any single measure. Future research should emphasize clearer construct definitions, longitudinal validation, fairness, and evaluation of emerging technologies.

## Introduction

Professionalism and empathy are foundational competencies in undergraduate medical education because they shape how future physicians interact with patients, colleagues, and healthcare systems. Professionalism generally encompasses ethical conduct, accountability, integrity, respect, reliability, and commitment to excellence, whereas empathy is more complex and variably defined across the literature. n medical education, empathy has been described as a multidimensional construct that includes cognitive understanding of a patient’s perspective, affective attunement or empathic concern, and the communication of that understanding in a way that patients can perceive [6, 8, 13].This distinction is important because internal empathy, self-reported empathic orientation, and observed empathic behavior are related but not interchangeable constructs.

These competencies are educationally and clinically important. More empathic and professional care has been associated with better patient experience and improved therapeutic relationships, and professionalism lapses during training have been linked to later disciplinary action in practice [5, 16]. At the same time, a recurrent concern in medical education is that empathy may decline during training, particularly during the transition from preclinical to clinical learning environments [9, 12]. Interpreting such findings, however, depends heavily on how empathy is measured. A decline in self-reported empathy does not necessarily indicate a parallel decline in observed empathic behavior, just as high professionalism ratings do not necessarily capture a learner’s internal empathic orientation.

This measurement problem is central to assessment. Over the past two decades, medical educators have used a wide range of tools to assess professionalism and empathy, including self-report instruments, faculty and standardized patient ratings, multisource feedback, objective structured clinical examinations (OSCEs), situational judgement tests, reflective portfolios, and more recently, technology-enhanced and artificial intelligence (AI)-supported approaches. These methods differ not only in format, but also in the constructs they target, the perspectives they capture, and the types of validity evidence available to support them. Self-report scales may provide efficient and psychometrically structured information about attitudes or orientation, whereas observer-based and patient-perceived measures assess behavior in context. Reflective assessments may reveal developmental insight, while AI-supported tools remain exploratory and require further validation.

For clarity, this review distinguishes three related constructs. Cognitive empathy refers to perspective taking and understanding; affective empathy or empathic concern refers to emotional attunement and concern; and empathic behavior refers to the observable interpersonal acts through which empathy is communicated and recognized in clinical encounters. That distinction is essential because commonly used measures in medical education target different levels of analysis. The Jefferson Scale of Empathy emphasizes clinical perspective taking, the Interpersonal Reactivity Index separates cognitive and affective dimensions more explicitly, and instruments such as CARE and the JSPPPE capture patient- or standardized-patient perceptions of empathic behavior during a specific encounter.

Although prior reviews have examined aspects of professionalism assessment or empathy measurement, many have focused on a single construct, a limited range of assessment modalities, or a narrower time frame. A broader synthesis that examines both professionalism and empathy together in undergraduate medical education, while distinguishing among the constructs being measured, remains needed. Such a synthesis is particularly relevant for educators designing assessment systems that must balance psychometric rigor, fairness, feasibility, and educational impact.

This systematic review therefore examines how professionalism and empathy have been assessed in undergraduate medical students from 2000 to 2025. The review focuses on the assessment methods used, the constructs they appear to measure, and the available evidence regarding reliability, validity, and educational utility. It also considers the implications of these findings for multimodal and programmatic assessment in undergraduate medical education.

## Methods

This systematic review was conducted in accordance with PRISMA guidance. The review examined published literature from January 2000 through October 2025 addressing the assessment of professionalism and/or empathy in undergraduate medical students.

### Search Strategy

A structured literature search was conducted in PubMed, ERIC, and Google Scholar using combinations of terms related to professionalism, empathy, medical students, undergraduate medical education, assessment, validity, reliability, OSCE, standardized patient, situational judgement test, multisource feedback, portfolio, Jefferson Scale of Empathy, and Professionalism Mini-Evaluation Exercise. Reference lists of key papers were also reviewed to identify additional relevant studies.

#### Eligibility Criteria

Studies were eligible for inclusion if they:

1. involved undergraduate medical students;
2. examined an assessment method or tool related to professionalism, empathy, or both; and
3. reported empirical findings relevant to measurement, validity, reliability, feasibility, or educational outcomes.

Included studies encompassed instrument development and validation studies, observational studies, and intervention studies in which professionalism or empathy assessment was an outcome. English-language articles were included. Editorials, opinion pieces, non-empirical commentaries, studies focused exclusively on residents or practicing physicians, and studies that discussed teaching without assessment data were excluded.

#### Study Selection

Titles and abstracts were screened for relevance, followed by full-text review of potentially eligible articles. Reviewer disagreements, where applicable, were resolved through discussion and consensus. The study selection process is summarized in the PRISMA flow diagram.

#### Data Extraction

Data were extracted on study characteristics, including country, learner level, sample size, assessment modality, target construct, and key findings related to reliability, validity, feasibility, and educational use. Particular attention was paid to whether an assessment method primarily captured self-reported orientation, observed behavior, patient- or standardized patient–perceived empathy, or professionalism-related conduct.

#### Quality Appraisal and Synthesis

Because the included studies varied substantially in design, setting, target construct, outcome definition, and assessment modality, meta-analysis was not appropriate. Findings were therefore synthesized narratively. Studies were grouped by assessment modality and interpreted in relation to the construct assessed. Methodological quality was appraised descriptively using features relevant to interpretation, including sample size, validation approach, reliability evidence, and clarity of measurement. The synthesis focused on cross-study patterns rather than exhaustive description of individual instruments.

#### PRISMA Flow

**Fig. 1:**
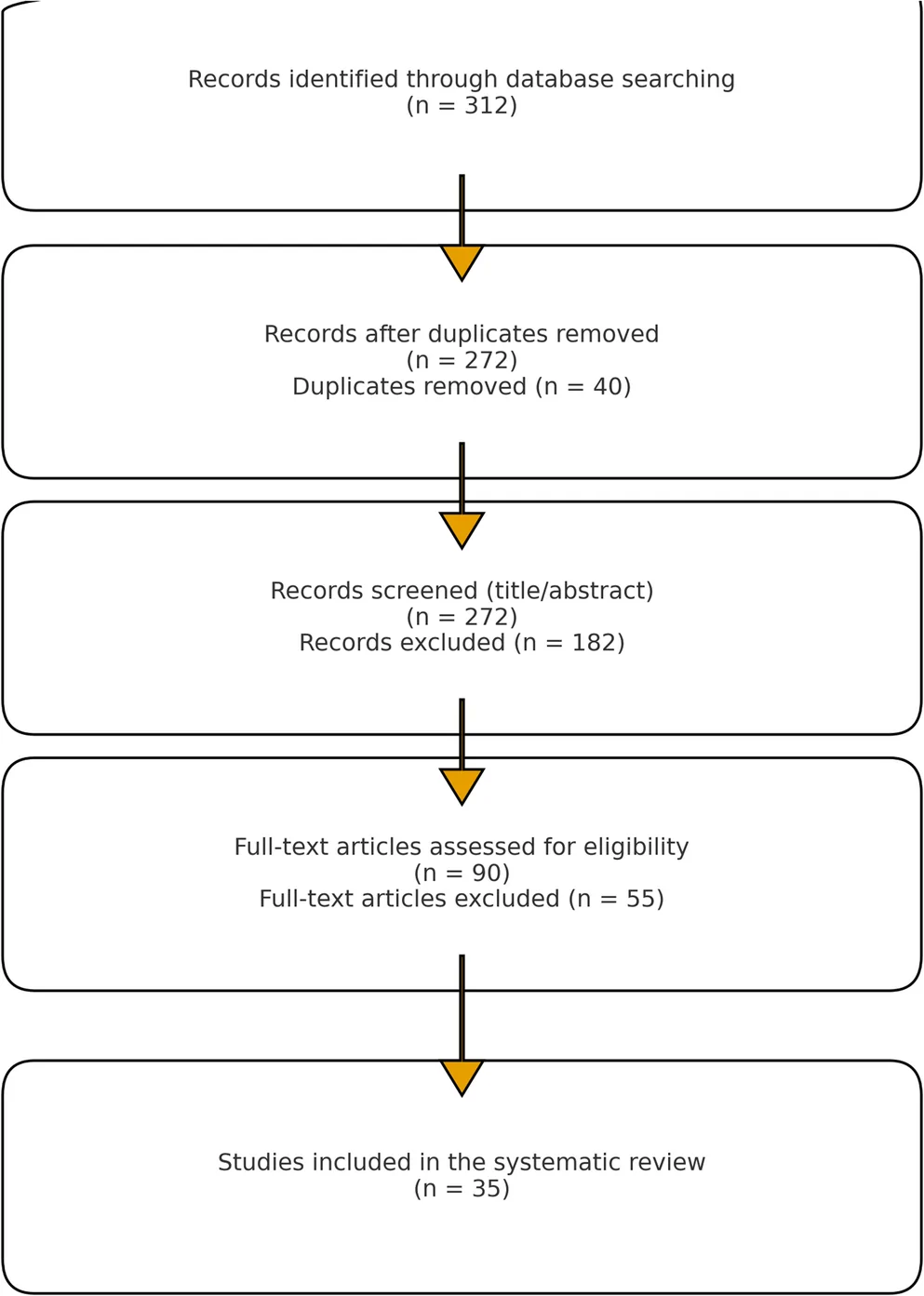
PRISMA 2020 flow diagram illustrating the selection of studies for the systematic review of professionalism and empathy assessment in medical students (2000–2025)

The literature search yielded 312 records. After removal of duplicates (*n* = 40), 272 unique titles/abstracts were screened. I excluded 182 at this stage (mostly irrelevant topics or wrong population). Ninety full-text articles were assessed for eligibility. Of these, 55 were excluded for reasons such as not focusing on assessment (narrative discussions without data) or involving postgraduate learners. Ultimately, 35 studies met all criteria and were included in the review (Fig. 1). Figure 1 presents the PRISMA 2020 flow diagram summarizing the study selection process. All included studies involved undergraduate medical students; a few also included comparison groups (e.g., residents or other health professions), but our synthesis focuses on findings for medical students.

## Results

The search yielded 312 records, of which 35 studies met inclusion criteria after screening and full-text review. The included literature spanned 2001 to 2025 and represented a range of study designs, including instrument development and validation studies, cross-sectional surveys, observational studies, performance-based assessments, and a smaller number of qualitative or mixed-methods studies. Most studies focused exclusively on undergraduate medical students, although a minority included mixed learner groups; in those cases, the synthesis focused on the medical student data reported in the study.

Across the included studies, assessment methods clustered into several broad modalities: self-report instruments, patient- or standardized patient–perceived measures, observer-based professionalism assessments, simulation- and scenario-based methods, reflective or narrative assessments, and a small number of emerging technology-enhanced approaches. A consistent finding across the literature was that these methods did not assess a single unified construct. Rather, they sampled related but distinct domains, including self-reported empathic orientation, multidimensional empathy traits, patient-perceived empathy in a specific encounter, observed professional behavior, and developmental or reflective aspects of professional formation [8, 14]. For that reason, results are organized by assessment modality and interpreted in relation to the construct each method most directly targets (Table 1).

**Table 1:** Summary of Assessment Modalities Used to Evaluate Professionalism and Empathy in Undergraduate Medical Education

Assessment modality	Common examples	Primary construct assessed	Main strengths	Main limitations	Suggested role in programmatic assessment
Self-report instruments	Jefferson Scale of Empathy (JSE), Interpersonal Reactivity Index (IRI), Professionalism Assessment Scale (PAS), Learners’ Attitude of Medical Professionalism Scale (LAMPS)	Empathic orientation, attitudes, self-perceived professionalism, beliefs and values	Efficient, scalable, easy to administer to large cohorts, often supported by internal consistency and factor-analytic data	Vulnerable to social desirability bias, ceiling effects, and limited self-insight; do not directly assess behavior	Best used for baseline assessment, longitudinal monitoring, and formative reflection rather than as stand-alone evidence of performance
Observer-based workplace assessments	Faculty clinical ratings, Professionalism Mini-Evaluation Exercise (P-MEX), multisource feedback	Observed professionalism, communication, teamwork, enacted empathic behavior in clinical settings	Capture authentic workplace performance and provide behaviorally grounded feedback	Subject to rater bias, halo effects, leniency, and context dependence; standards may vary across raters and settings	Best used as repeated low-stakes observations aggregated over time across multiple raters
Patient- or standardized patient–perceived measures	CARE measure, standardized patient empathy ratings, patient feedback forms	Perceived empathy during a specific encounter	Reflect recipient experience and therefore capture whether empathy was actually perceived	Encounter-specific, influenced by case characteristics and rater expectations, less suitable as a trait-level measure	Valuable as a complementary perspective alongside self-report and faculty observation
Simulation- and scenario-based assessments	Objective structured clinical examinations (OSCEs), situational judgement tests (SJTs)	Communication, ethical reasoning, professional judgment, observable empathic behavior under standardized conditions	More standardized than workplace ratings; useful for comparing learners under similar conditions	Artificiality, station specificity, and limited generalizability to routine clinical performance	Useful as structured checkpoints within a broader multimodal assessment system
Reflective and narrative assessments	Portfolios, reflective essays, narrative evaluations	Reflective capacity, professional identity formation, self-awareness, developmental insight	Capture longitudinal growth and meaning-making; support coaching and professional formation	Time intensive, potentially subjective, and less appropriate as isolated summative measures	Best used formatively and interpreted alongside other quantitative and observational data
Emerging technology-enhanced approaches	AI-supported communication analysis, virtual patients, chatbot-based simulations	Exploratory indicators of communication, empathy-related language, and interaction patterns	Scalable, potentially efficient, and able to generate rapid feedback	Limited validity evidence, fairness concerns, unclear construct boundaries, and possible loss of human context	Currently best used as supplementary formative tools rather than high-stakes assessment methods

Assessment methods differed not only by format but also by the construct targeted. Self-report instruments primarily assessed attitudes or orientation, whereas patient- or standardized patient–based measures assessed perceived empathy in a specific encounter, and workplace-based tools assessed enacted behavior in context. These modalities should therefore be interpreted as complementary rather than interchangeable

### Self-Report Instruments

Self-report measures were the most frequently represented assessment modality and were used primarily to assess empathic orientation, trait-like empathy, professionalism attitudes, or self-perceived professional values rather than directly observed behavior.

The Jefferson Scale of Empathy (JSE) was the most commonly cited empathy instrument. Across the reviewed studies, it showed the strongest and most consistent psychometric support among self-report empathy measures, including favorable internal consistency and broad use in undergraduate medical education [7, 8]. The JSE was particularly useful for assessing clinically oriented empathic perspective taking and attitudes toward empathic patient care. National normative work in medical students reported mean scores around 116.5 with Cronbach’s α approximately 0.82, supporting its use as a stable self-report instrument in this population [7]. Some Jefferson studies also examined good-impression responding using external infrequency measures, highlighting the relevance of social desirability bias in self-report assessment rather than indicating that the JSE directly measures enacted empathy [7].

A recurrent finding in JSE-based studies was modest decline in self-reported empathy during clinical training, although interpretation of this pattern requires caution because the measure captures self-perceived empathic orientation rather than observed empathic behavior [9, 12]. This distinction was important across the review: lower self-reported empathy over time does not necessarily imply parallel decline in patient-perceived or observer-rated empathic behavior.

The Interpersonal Reactivity Index (IRI) appeared less often but was conceptually important because it explicitly separates empathy into subdomains such as perspective taking, empathic concern, personal distress, and fantasy [4]. In the included studies, the IRI helped illustrate that empathy is multidimensional and that cognitive and affective components should not be collapsed into a single construct. Its main limitation in this literature was that it was not designed specifically for medical contexts and therefore offered less direct relevance to clinical performance than the JSE. Several studies also used self-report professionalism instruments, most notably the Professionalism Assessment Scale (PAS) and the Learners’ Attitude of Medical Professionalism Scale (LAMPS). These tools generally demonstrated acceptable internal consistency and were useful for assessing professionalism-related attitudes, values, and self-concept [1, 11]. However, the same limitations seen in empathy self-report measures applied here as well: they are efficient and scalable, but vulnerable to ceiling effects, social desirability, and limited ability to establish whether endorsed values are enacted consistently in practice.

Overall, the self-report literature supported these measures as useful tools for baseline assessment, longitudinal monitoring, and formative reflection. Their principal limitation was that they provided evidence about orientation, beliefs, or self-perception rather than direct evidence of observed behavior in clinical settings [4, 7].

### Patient, Standardized Patient, and Observer-Based Assessments

Observer-based and recipient-based methods assessed professionalism and empathy as enacted or perceived in the context of clinical or simulated encounters. These approaches were particularly important because they moved beyond self-description to address how behavior was experienced by others. Patient- and standardized patient–perceived measures, particularly the Consultation and Relational Empathy (CARE) measure, assessed whether empathy was recognized by the recipient in a specific encounter. In its original validation, CARE demonstrated high internal consistency and strong relational focus, supporting its use as an encounter-level measure of perceived empathy (Cronbach’s α ~ 0.96–0.97 in some studies) [14]. Within the reviewed literature, these measures contributed a perspective that self-report instruments could not provide: whether empathy was actually experienced by the patient or SP. At the same time, their interpretation remained encounter-bound. Ratings could vary as a function of case content, patient expectations, communication style, and the local context of the interaction. These measures therefore were best understood as indicators of perceived empathy in context rather than stable trait-level evidence.

Observer-based professionalism measures, including faculty ratings and the Professionalism Mini-Evaluation Exercise (P-MEX), captured visible professional conduct in clinical settings. The P-MEX was among the strongest observational professionalism tools in the reviewed literature and showed favorable validity evidence, including strong internal consistency (α ≈ 0.91) and a reproducible multidomain structure encompassing doctor–patient skills, interprofessional conduct, professional demeanor, and reflective ability [2]. Its main contribution was to operationalize professionalism through specific observable behaviors. However, the review also indicated that professionalism-focused observational tools should not be treated as direct measures of internal empathy. Their relevance to empathy lies primarily in overlap with visible empathic conduct, not in direct access to cognitive or affective empathic orientation.

Multisource feedback expanded the observational perspective by incorporating input from multiple raters, including faculty, peers, and in some cases patients. The reviewed literature suggested that multisource systems improved breadth and reliability when enough observers contributed ratings. For example, aggregated multisource feedback showed acceptable reliability when ratings were collected from multiple evaluators, supporting its use for professionalism assessment across settings [15]. Notably, the generalizability coefficient (a reliability measure) was around 0.7 with 8 raters, improving to > 0.8 with ≥ 12 raters, indicating that more raters yield more reliable composite scores. This was particularly valuable for behaviors such as teamwork, accountability, and respectful interaction, which may not be fully visible to a single evaluator. Across these studies, convergence between self-report and observed or perceived performance was limited. Earlier work found that self-reported empathy was associated with academic and clinical performance, but later observational literature suggested that self-report and encounter-based empathy ratings often aligned only weakly [8, 14]. This limited convergence was one of the most important findings in the review. Rather than implying that one modality was invalid, it suggested that different tools were sampling different facets of empathy and professionalism: self-report measured orientation, patient/SP ratings measured experienced empathy, and faculty/observer ratings measured visible conduct judged in professional context.

### Simulation- and Scenario-Based Assessments

Simulation-based assessments, including objective structured clinical examinations (OSCEs) and situational judgement tests (SJTs), provided a more standardized approach to assessing communication, professionalism, ethical reasoning, and observable empathic behavior.

OSCE-based assessments allowed direct observation of students’ responses to structured interpersonal and professional challenges. In the reviewed studies, OSCEs were particularly useful for assessing behavioral manifestations of empathy, such as acknowledging emotion, listening responsively, and communicating concern, as well as professionalism-related decision-making in ethically complex situations [8]. Their primary strength was comparability: learners could be assessed under similar conditions using common scoring criteria. However, the literature also emphasized their limitations, including artificiality, station dependence, and uncertain generalizability to routine clinical practice.

SJTs assessed decision-making in hypothetical scenarios involving professionalism, ethics, teamwork, and empathy-related judgment. The reviewed studies suggested that SJTs demonstrated acceptable psychometric performance and were especially useful for sampling responses to situations that are important educationally but difficult to observe consistently in real practice, such as dishonesty, fatigue-related judgment, or interpersonal conflict [10]. Their main limitation was that they measured stated judgment rather than actual behavior in a live clinical setting.

Taken together, simulation- and scenario-based methods occupied an intermediate position between standardization and authenticity. They offered more comparability than workplace observation but less ecological realism. The literature supported their use as one component of a broader assessment strategy rather than as stand-alone indicators of professionalism or empathy.

### Reflective and Narrative Assessments

A smaller subset of included studies examined portfolios, reflective writing, and other narrative assessments. These methods focused less on psychometric standardization and more on developmental insight, self-awareness, and professional identity formation. The reviewed literature suggested that reflective assessments were particularly useful for capturing how learners made sense of challenging clinical, ethical, or emotionally salient experiences. Portfolios and reflective narratives often revealed aspects of professionalism and empathy not readily captured through surveys or checklists, including awareness of bias, emotional processing, responsibility, and meaning-making over time [18]. These methods therefore contributed a distinct developmental perspective that complemented more structured measures.

Their limitations were also consistent across studies. Reflective assessments were time-intensive, difficult to standardize, and vulnerable to assessor subjectivity. For that reason, the literature supported their use primarily in formative and longitudinal contexts rather than as isolated summative measures. Their strongest role appeared to be within multimodal systems that combined narrative reflection with observational and psychometric data [18].

### Emerging Technology-Enhanced Approaches

Technology-enhanced and AI-supported approaches were represented only sparsely in the included literature. These studies explored virtual patients, automated communication analysis, and AI-assisted feedback on language or interviewing behavior relevant to empathy and professionalism.The limited available evidence suggested potential strengths in scalability, rapid feedback, and reproducibility. For example, AI-supported systems could enable repeated practice without the resource demands associated with faculty observation or standardized patients. However, the evidence base remained preliminary, and validity concerns were substantial. The reviewed literature identified unresolved questions related to construct definition, fairness, bias, and the risk of oversimplifying empathy into decontextualized linguistic or vocal markers [3, 17].Accordingly, the available evidence supported viewing AI-enhanced approaches as exploratory adjuncts rather than established methods for high-stakes assessment.

### Cross-Modal Synthesis

Across modalities, the literature consistently indicated that professionalism and empathy assessment in undergraduate medical education is not a single-measure problem. Self-report instruments assessed empathic orientation and professional attitudes; patient- and standardized patient–based measures assessed perceived empathy within specific encounters; observer-based tools assessed visible conduct; simulation-based methods assessed performance in standardized scenarios; and reflective approaches assessed developmental insight and professional identity work [2, 8, 14, 18]. A recurring pattern was limited convergence across these approaches. This pattern was most plausibly interpreted not as simple measurement failure, but as evidence that different modalities captured related but nonidentical constructs. The overall literature therefore supported a multimodal interpretation in which multiple forms of evidence are combined over time to produce a more defensible understanding of learner development than any single instrument could provide alone [2, 15] (Table 2).

**Table 2:** Practical Implications of Assessment Modalities for Programmatic Assessment of Professionalism and Empathy

Modality	What it contributes	Major source of bias or validity threat	Interpretation guidance	Practical recommendation for educators
Self-report	Information about learner attitudes, empathic orientation, and self-perceived professional values	Social desirability bias, ceiling effects, limited self-awareness	Should not be interpreted as direct evidence of professional or empathic behavior	Use for formative reflection, developmental monitoring, and triangulation with observed performance
Faculty and workplace ratings	Evidence of performance in authentic clinical environments	Halo effects, leniency, context dependence, variable evaluator expectations	Single ratings should be interpreted cautiously; patterns across repeated observations are more meaningful	Aggregate multiple ratings across rotations and evaluators before making higher-stakes judgments
Patient or standardized patient ratings	Recipient perspective on whether empathy and professionalism were perceived	Case specificity, rater expectations, response tendencies	Best interpreted as encounter-level evidence of relational effectiveness rather than stable trait evidence	Include as a distinct perspective within a multimodal assessment system
OSCEs and SJTs	Standardized evidence of communication, ethical reasoning, and professional decision-making	Artificiality, limited transfer to routine practice, station or scenario dependence	Strong for comparability, but insufficient alone to represent authentic longitudinal performance	Use as one component of an assessment portfolio rather than as the sole indicator of professionalism or empathy
Portfolios and reflective writing	Evidence of reflective capacity, insight, and professional identity development	Performative reflection, assessor subjectivity, scoring burden	Most useful for developmental interpretation rather than isolated summative decisions	Pair with coaching, narrative feedback, and longitudinal review
AI-supported tools	Scalable supplementary feedback and pattern recognition in communication	Algorithmic bias, unclear construct validity, reduced contextual nuance	Should be interpreted conservatively until stronger validation evidence is available	Restrict to exploratory or formative use at present

Programmatic assessment emphasizes the aggregation of multiple low- and moderate-stakes data points over time. In this framework, disagreement across modalities should not automatically be viewed as a flaw; it may indicate that different tools are capturing different but relevant dimensions of professionalism and empathy

## Discussion

This review indicates that professionalism and empathy in undergraduate medical education are assessed through multiple modalities that provide complementary, but not interchangeable, forms of evidence. A central finding is that variability across measures does not necessarily indicate methodological weakness; in many cases, it reflects differences in the constructs being assessed. Self-report tools primarily capture empathic orientation, attitudes, or self-perception rather than directly observed behavior [7, 8]. Patient- and standardized patient–based measures capture empathy as experienced within a specific encounter, whereas faculty ratings, workplace-based assessments, and professionalism instruments capture observed conduct in context [2, 14–16]. These approaches therefore should not be expected to converge perfectly, even when each is functioning appropriately.

A second major finding is that no single method adequately captures the complexity of either professionalism or empathy. Within the reviewed literature, relatively stronger psychometric support was reported for established self-report tools such as the Jefferson Scale of Empathy and for structured observational tools such as the P-MEX [2, 7]. However, psychometric strength alone does not resolve the broader validity question, because reliability and internal consistency do not guarantee that a measure captures authentic clinical behavior. Conversely, assessments that may appear more authentic, such as faculty ratings, standardized patient feedback, and portfolios, can offer valuable contextual information but are more vulnerable to rater effects, case specificity, and interpretive variability [14, 15, 18].

### Why Modalities Diverge

The divergence among measures is educationally important. Empathy is often discussed as though it were a single trait, but the reviewed studies suggest that self-reported empathy, observed empathic behavior, and patient-perceived empathy represent overlapping but distinct domains [8, 14]. Professionalism presents a similar challenge because it includes multiple elements, including ethical conduct, accountability, communication, teamwork, and reliability, that may not be equally visible in every setting [2]. As a result, disagreement across modalities should not automatically be interpreted as evidence that one tool is invalid or that another is superior. In many cases, it indicates that the tools are sampling different dimensions of performance. This helps explain why self-report measures may identify broad developmental trends, while observer-based or encounter-based measures yield more context-specific judgments. A learner may endorse empathic values on a questionnaire yet struggle to communicate empathy effectively under time pressure. Conversely, a learner may perform well behaviorally in a structured encounter without reporting especially high empathic orientation on self-assessment. Earlier studies also reported only limited convergence between self-reported empathy and observed or perceived empathy, reinforcing the view that cross-modal disagreement may reflect construct differences rather than simple measurement failure [8, 14]. These findings reinforce the need for construct clarity when interpreting assessment results.

### Trade-Offs Across Assessment Modalities

The reviewed literature suggests a consistent trade-off between standardization and authenticity. Self-report scales and some standardized scenario-based methods offer efficiency, comparability, and clearer psychometric structure, making them useful for large cohorts and longitudinal benchmarking [7, 10]. However, they are limited by social desirability bias, ceiling effects, and the fact that they assess perception or judgment rather than actual behavior [7]. Observer-based ratings, workplace-based assessments, and multisource feedback provide more direct evidence of enacted professionalism and empathy, but they are subject to halo effects, leniency, inconsistent standards, and context dependence [2, 15]. Simulation-based assessments such as OSCEs and situational judgement tests occupy an intermediate position. They provide more standardization than workplace-based assessments while allowing evaluation of communication, ethical reasoning, and professional decisionmaking under controlled conditions [8, 10]. However, they remain bounded by case design and may not fully generalize to routine clinical work. Reflective portfolios and narrative assessments contribute a developmental perspective, particularly for professional identity formation and reflective capacity, but they are less suited to stand-alone summative judgments because of subjectivity and scoring burden [18].

### Implications for Programmatic Assessment

These findings support a programmatic assessment approach in undergraduate medical education. This interpretation follows directly from the limited convergence observed across self-report, patient/SP, and observer-based measures in the reviewed studies [2, 10, 16]. If different methods capture different aspects of professionalism and empathy, then educationally defensible judgment should rely on the aggregation of multiple low- and moderate-stakes data points over time rather than on a single score or encounter. Programmatic assessment is particularly relevant for complex domains that include attitudes, communication, professional conduct, and contextual performance.

In this framework, self-report instruments may help identify learner orientation and developmental trends; structured observational tools may capture performance in authentic settings; standardized patient or patient-reported measures may contribute recipient perspectives; and reflective assessments may provide insight into growth and self-awareness [2, 7, 14, 18]. This multimodal approach also has practical implications for feedback and coaching. Rather than treating discrepancies across measures as failure, educators can use them diagnostically. For example, divergence between high self-reported empathy and weaker patient-perceived empathy may identify a communication gap rather than a lack of caring intent. Similarly, strong professionalism ratings in one setting but not another may suggest context-specific challenges rather than a generalized deficit. In this sense, programmatic assessment can support both learner development and more defensible decision-making.

### Bias, Fairness, and Future Directions

The review also highlights important validity and fairness concerns. Self-report measures are vulnerable to social desirability and limited self-insight [8]. Observer-based ratings may be influenced by interpersonal fit, communication style, demographic bias, and inconsistent expectations across raters and contexts [2, 15]. These concerns suggest that assessment systems should distinguish core professional obligations from superficial stylistic conformity and should rely on clear criteria, repeated sampling, and multiple perspectives. This issue is especially relevant when assessing learners whose communication styles differ from dominant norms, including neurodivergent learners and students from diverse cultural backgrounds. Although the direct evidence base on neurodiversity in empathy and professionalism assessment remains limited in the reviewed studies, the broader fairness literature supports caution in how normative judgments are operationalized and interpreted [19]. Emerging AI-supported approaches remain promising but preliminary. The reviewed literature suggests potential value in scalability, rapid feedback, and analysis of communication patterns, but the evidence base remains limited [3, 17]. AI tools should therefore be viewed as supplementary rather than replacement methods until stronger validity, fairness, and educational impact data are available. Several gaps in the literature remain. First, more longitudinal work is needed to understand how professionalism and empathy develop over time and how different assessment modalities relate to later performance [9, 16]. Second, clearer construct definitions are needed in future studies so that findings can be interpreted with greater precision [8]. Third, research should examine how assessment systems can support fairness while maintaining authentic evaluation of communication and professional behavior [19]. Overall, the reviewed evidence suggests that multimodal assessment is not simply a pragmatic compromise; it is a conceptually necessary response to the multidimensional nature of professionalism and empathy in medical education.

## Conclusion

This review indicates that professionalism and empathy in undergraduate medical education cannot be captured adequately by any single assessment method. Self-report instruments, patient- and standardized patient–perceived measures, observer-based ratings, simulation-based assessments, and reflective approaches each provide distinct but incomplete evidence. Their differences are not simply methodological flaws; they reflect the fact that these tools assess related but nonidentical constructs.

The main contribution of this review is to clarify that assessment of professionalism and empathy is best approached as a multimodal and programmatic process rather than a search for one definitive instrument. For educators, the practical implication is clear: assessment systems should combine complementary methods, interpret results with attention to construct and context, and use multiple observations over time to guide feedback, coaching, and high-stakes decisions. Future research should prioritize clearer construct definitions, stronger longitudinal validation, and careful evaluation of newer technologies within authentic educational settings.
